# A UK survey of driving behaviour, fatigue, risk taking and road traffic accidents

**DOI:** 10.1136/bmjopen-2016-011461

**Published:** 2016-08-18

**Authors:** Andrew P Smith

**Affiliations:** Centre for Occupational and Health Psychology, School of Psychology, Cardiff University, Cardiff, UK

**Keywords:** road traffic accidents, driving behaviour, fatigue, risk taking

## Abstract

**Objective:**

The aim of the present research was to examine associations between poor driving behaviour (DB), driving when fatigued (DF), risk taking (RT) and road traffic accidents (RTAs).

**Design:**

The study involved a cross-sectional online survey of clients of an insurance company. The survey measured DB (speeding, distraction, lapses of attention and aggression), RT and frequency of driving when fatigued (DF, driving late at night, prolonged driving, driving after a demanding working day and driving with a cold). Demographic, lifestyle, job characteristics and psychosocial factors were also measured and used as covariates.

**Setting:**

Cardiff, UK.

**Sample:**

3000 clients of an insurance company agreed to participate in the study, and 2856 completed the survey (68% woman, 32% man; mean age: 34 years, range 18–74 years).

**Main outcome measures:**

The outcomes were RTAs (requiring medical attention; not requiring medical attention), where the person was the driver.

**Results:**

Factor analyses showed that DB, RT and fatigue loaded on independent factors. Logistic regressions showed that poor DB, frequently DF and taking risks predicted medical and non-medical RTAs. These effects were additive and those who reported poor DB, driving when fatigue and taking risks were twice as likely to have an RTA. These effects remained significant when demographic, lifestyle, medical, driving, work and psychosocial factors were covaried.

**Conclusions:**

Poor DB, DF and RT predict RTAs. There are now short measuring instruments that can assess these, and driver education programmes must increase awareness of these risk factors.

**Strengths and limitations of this study**

- This survey identified poor driving behaviour, driving when fatigued and risk taking as predictors of road traffic accidents.
- It controlled for personal and occupational factors.
- It used short measuring instruments that can be used in risk assessments.
- The results have implications for information campaigns and training.
- The survey was cross-sectional, which makes it difficult to assess causality.

## Introduction

Road traffic accidents (RTAs) are a major cause of mortality, injury and financial cost,^1^ and it is generally acknowledged that human error is frequently involved.^2^ There has been considerable research on risk factors for RTAs, and legislation aims to prevent some effects (eg, effects of alcohol and drugs). Other issues such as fatigue are often addressed in professional drivers^3^ and the general public.^4^ Driver fatigue is now considered to be a major contributor to 15–30% of all crashes.^4–8^ Inappropriate driving behaviour (DB) (eg, speeding) is often dealt with by sanctions and/or by attendance at appropriate training courses.^9^ A major problem with much of the research is that factors are often studied in isolation whereas it is clear that a multivariate approach is essential. This is true for the risk factors and the outcomes. In addition, it is important to adjust for possible confounders, which may influence risk factors and outcomes (eg, demographic variables, lifestyle, job characteristics and psychosocial factors). In order to conduct such research, it is important to develop short measuring instruments that collect data on a wide range of variables. This approach has been used to address issues such as well-being^10^ and can now be applied to driver safety.

The potential risk factors considered here were poor DB, driving when fatigued (DF) and risk taking (RT). Driver behaviour has frequently been assessed by the questionnaire,^11^ ^12^ and four types of problem that have been identified are speeding, errors, lapses of attention and aggressive driving. Our previous research^13^ has shown that single items measuring these aspects of DB are highly correlated with the overall scales, and these were used in the present study. RT is a general type of behaviour, which becomes important in safety critical contexts such as driving. Again, we have developed single questions measuring RT at work and outside work,^13^ which correlate highly with the DOSPERT Scale.^14^ These single items were used in the present study. What is unclear is whether RT reflects other factors such as driver behaviour or fatigue. RT is known to increase when people are fatigued,^15^ but it may also reflect other characteristics such as personality.^16^

Much of the research on driver fatigue has focused on the length of time spent driving.^17^ However, fatigue may be due to many factors, and there is evidence that driving impairments are related to time of day,^6^ loss of sleep or sleep inertia,^18^ prolonged work^19^ and minor illness.^20^ It is important, therefore, to assess the frequency with which people drive when they are potentially fatigued because of this range of factors. One objective of the present study was to develop single questions measuring driving in different fatigue states. Analyses were then conducted to determine whether these items formed a single factor or were independent. The analyses also examined whether DB, DF and RT were related or independent.

One problem with previous research on driving is that it often fails to take a multivariate approach. There is substantial evidence that demographic factors are related to RTAs (eg, young men are known to be at a greater risk of having an accident^21^). Similarly, lifestyle factors such as alcohol use are established risk factors for impaired driving.^22^ Other research has shown that job characteristics such as working at night, doing shift work or working prolonged hours increase the risk of an RTA.^23^ In addition, work-related outcomes such as job stress may be associated with impaired driving.^24^ Psychological characteristics such as personality have also been associated with the risk of having an RTA.^25^ The present study provided an opportunity to conduct multivariate analysis based on a range of different variables. This allowed examination of whether the primary variables were still associated with the outcomes when other factors were covaried. It also allowed bench marking of the effects of the different types of variable.

## Aims and method

The aims and objectives of the present study were to use a multivariate approach to examine associations between reported driver behaviour, driving when potentially fatigued, RT and RTAs while adjusting for possible confounding factors (demographics, lifestyle, job characteristics and psychosocial factors).

### Method

The study was carried out with the approval (EC.16.6.06) of the ethics committee, School of Psychology, Cardiff University and the informed consent of the volunteers.

### Participants

A sample size calculation suggested that a sample of 2000 would be appropriate to detect effects of the potential risk factors after adjustment for multiple covariates. Clients of an insurance company^26^ who were in current employment and had agreed to receive communications from the company were sent information about the study. Those who were willing to participate were sent a link to the online survey. This continued until 3000 volunteers had expressed an interest in participating in the study. Of those, 2856 (95.2%) completed the survey. Details of the final sample are shown in table 1.

**Table 1:** Characteristics of the final sample

Gender	68% female
Age	Mean age 34 years; range 18–74 years
Married/living with partner	61.2%
Education	55.5% degree/professional qualification; 24.5% ‘A level’; 20.4% GCSE
Salary	10.6%<£10 000 pa; 38.8% £10–25 000 pa; 29.6% £25–40 000 pa; 19.6%>£40 000 pa
Full-time job	87.9%
Permanent job	89.3%
Type of job	8% self-employed; 23.3% managers; 10.4% supervisors; 58.3% employees.

### The survey

The survey was described as being about driving and associated behaviour. It was administered using Survey Tracker software. It consisted of several sections:

1. The first section was about driving. This contained the questions on poor DB^7^ and driver fatigue (see box 1). It also contained questions about frequency of driving on motorways and in heavy traffic, frequency of driving in bad weather and ratings of driving ability.
2. The second section was about the nature of the person's job and the questions were taken from the Bristol Stress and Health at Work Survey.^27^ They covered type of job, working hours, work environment and psychosocial job characteristics (demands, control, support, effort/reward imbalance). In addition, job satisfaction, stress at work and work–life balance were assessed.
3. The third section assessed physical and mental health (chronic health problems, acute symptoms, anxiety and depression) and use of medication.^27^
4. The fourth sections covered accidents, injuries and cognitive failures at work and outside work. The RTA questions were in this section (see box 1).^28^
5. The fifth section asked questions about lifestyle (alcohol consumption, smoking and diet). The RT questions were in this section (see box 1).^28^
6. The final section provided the demographic, personality^29^ and insurance behaviour information.

Box 1 **Questions measuring driving behaviour, driver fatigue, risk taking and road traffic accidents**

*Driving behaviour:*

- How often do you have lapses of concentration when driving?
- How often do you indicate hostility to other drivers?
- How often do you miss warning signs?
- How often do you disregard the speed limit on a residential road?

*Driver fatigue:*

- How often do you have to drive when you are tired?
- How often do you drive when you have a minor illness like a cold?
- How often do you have to drive late at night, in the early morning or the postlunch period?
- How often do you have to drive for long periods?
- How often do you have to drive after prolonged work?

*Risk taking:*

- How frequently do you take risks at work?
- How frequently do you take risks outside of work?

Responses to the above questions made on a five-point scale: 0=Never; 1=Rarely; 2=Sometimes; 3=Often; 4=Very Often

Road traffic accidents:

Thinking about the last 12 months, have you been involved in any traffic accidents when you have been the driver resulting in injuries that required medical attention from someone else (eg, a first aider, GP, nurse or hospital doctor)?

Number:

Thinking about the last 12 months, have you been involved in any traffic accidents when you have been the driver that have not involved injuries?

Number:

### Statistical analysis plan

Factor analyses were carried out on the driver behaviour, driver fatigue and RT questions to determine if they were independent. Univariate logistic regressions were then carried out examining associations among DB, DF, RT and RTAs. Multivariate logistic regressions were then carried out with demographics, job characteristics, personality, driving and health variables as covariates. Combined effects of the risk factors were then examined in another series of logistic regressions by adding the scores from the median splits to give four groups: those with no risk factors, those with one, those with two and those with all three. This enabled one to examine dose–response. Analyses were carried out using IBM SPSS Statistics 20.

## Results

Factor analysis (with a varimax rotation) of the DF, DB and RT questions showed that these variables loaded on separate factors (see table 2) The Cronbach αs for the three factors were 0.78 (DF), 0.75 (DB) and 0.72 (RT).

**Table 2:** Factor analysis of fatigue, DB and RT questions (sorted rotated component matrix; loadings<0.3 not shown)

	Fatigue factor: Eigenvalue=3.357 % variance=30.52	DB factor: Eigenvalue=1.50 % variance=13.62	RT: Eigenvalue=1.33 % variance=12.14
Drive late at night	0.774		
Drive after prolonged work	0.774		
Drive for long periods	0.734		
Drive when tired	0.638		
Drive with a cold	0.470		
			
Lapses of concentration		0.794	
Speeding		0.747	
Miss warning signs		0.687	
Hostility to others		0.434	
Risks at work			0.865
Risks outside work			0.860

DB, driving behaviour.

The factor scores were used in the analyses of the RTAs. Initially, each factor score was split at the median and low and high groups compared. There were more accidents not requiring medical attention (10.4%) than those that did (4.7%). Univariate logistic regression showed that DB, DF and RT were significant predictors of both types of accident (see table 3).

**Table 3:** Univariate logistic regressions with RTAmed and RTAnomed as outcomes

	OR	CIs	Significance	Omnibus test of model coefficients (χ^2^)
DB and RTAmed	1.73	1.21 to 2.49	p<0.01	9.23, p<0.005
DB and RTAnomed	1.61	1.26 to 2.06	p<0.01	14.59, p<0.001
Fatigue and RTAmed	1.63	1.14 to 2.32	p<0.01	7.26, p<0.01
Fatigue and RTAnomed	1.70	1.32 to 2.18	p<0.001	17.8, p<0.001
RT and RTAmed	1.63	1.14 to 2.33	p<0.01	7.49, p<0.01
RT and RTAnomed	1.40	1.10 to 1.79	p<0.05	7.57, p<0.01

DB, driving behaviour; RT, risk taking; RTA, road traffic accident; RTAmed, RTA requiring medical attention; RTAnomed, RTA requiring no-medical attention.

The next set of analyses included DB, DF and RT in the same analyses (using the ENTER method), and all of the effects remained significant (see table 4).

**Table 4:** Multivariate logistic regressions including DB, driver fatigue and RT as predictor variables with RTAmed and RTAnomed as outcomes

	OR	CIs	Significance	Omnibus test of model coefficients (χ^2^)
RTAmed				20.04, p<001
DB	1.61	1.12 to 2.32	p<0.05	
Fatigue	1.48	1.03 to 2.14	p<0.05	
RT	1.49	1.04 to 2.13	p<0.05	
RTAnomed				33.87, p<0.001
DB	1.51	1.18 to 1.95	p<0.005	
Fatigue	1.60	1.24 to 2.05	p<0.05	
RT	1.28	1.01 to 1.63	p<0.05	

DB, driving behaviour; RT, risk taking; RTA, road traffic accident; RTAmed, RTA requiring medical attention; RTAnomed, RTA requiring no-medical attention.

Analyses of the demographic, personality, health, driving and job variables showed that accidents were predicted by being single/divorced/separated, driving frequently in bad weather, being rated as a poor driver, taking psychotropic medication and having a job with a lot of negative characteristics (high demands, low control/support, poor working environment, shiftwork/long working hours). These variables were included as covariates in logistic regression examining both types of RTA and the effects of DB, DF and RT remained significant (see table 5).

**Table 5:** Logistic regressions including covariates, DB, driver fatigue and RT with the combined RTA score as the outcome

	OR	CIs	Significance	Omnibus test of model coefficients (χ^2^)
				58.4, p<0.001
DB	1.36	1.05 to 1.76	p<0.05	
Driver fatigue	1.55	1.18 to 2.04	p<0.005	
RT	1.28	1.01 to 1.64	p<0.05	
Marital status	1.31	1.02 to 1.68	p<0.05	
Being rated by others as a good driver	0.73	0.61 to 0.86	p<0.001	

DB, driving behaviour; RT, risk taking; RTA, road traffic accident.

Additional analyses examined possible threshold effects by splitting the DB, DF and RT variables into quartiles and examining associations with the combined RTA score. The effect of DB only became significant in the fourth quartile (OR=1.41, CI 1.05 to 1.90). In contrast, DF showed a clear linear trend, and quartiles 3 and 4 were significantly different from the first quartile (Q3: OR=1.61, CI 1.17 to 2.22; Q4: OR 1.70, CI 1.24 to 2.33). For RT, Q3 and Q4 were different from Q1 (Q3: OR=1.27, CI 0.93 to 1.75; Q4: OR=1.24, CI 0.91 to 1.67). Finally, the combined effects of DB, DF and RT were examined by adding the scores from the median splits to give four groups: those with no risk factors, those with one, those with two and those with all three. A clear dose–response was observed with risk of an RTA increasing linearly with the number of risk factors (see table 6).

**Table 6:** Logistic regression examining the combined effects of DB, driver fatigue and RT (split into quartiles) on the combined RTA score

	OR	CI	Significance	Omnibus test of model coefficients (χ^2^)
				20.94, p<0.001
No risk factors	1			
One risk factor	1.19	0.80 to 1.77	p>0.05	
Two risk factors	1.57	1.06 to 2.34	p<0.05	
All three risk factors	2.55	1.70 to 3.82	p<0.001	

DB, driving behaviour; RT, risk taking.

## Discussion

The results of this survey confirm that poor DB, DF and risk factors predict RTAs. These effects were still apparent when demographic, driving, lifestyle, health, psychosocial and work characteristics were covaried. The three risk factors produced additive effects with those who had all three being 2.55 times more likely to have an accident than those reporting no risk factors.

The present study clearly has some limitations, and further research is required to extend these. First, the sample was selected from clients of an insurance company rather than being representative of the general population. The online nature of the survey may also have excluded some individuals. The study was a cross-sectional survey, which makes it difficult to be confident about causality. However, the presence of clear dose–response relationships is some evidence for causal relationships. Future research should provide more information on the type of RTA (eg, single vehicle; multivehicle driver responsible) as there may be some noise in the present data due to the inclusion of accidents that were not due to the respondent.

The two key features of the present research were the use of short measuring instruments and control of a wide range of possible confounders. The audit tool could now be even shorter, and three questions reflecting DB, fatigue and RT could be asked (eg, How often do you drive inappropriately—eg, miss signals, have lapses of concentration, are hostile to other drivers, disregard the speed limit? How often do you drive for long periods or when you are tired—eg, late at night, in the early morning or the postlunch period, after prolonged work? How often do you take risks?). A major problem is that people may answer these honestly in an anonymous questionnaire but may not do this in other situations (eg, when taking out an insurance policy). However, objective information can verify some of these (eg, speeding offences or prior accident information is available), and mobile phone aps can give a good indication of when people drive.

It is clearly important to educate drivers to increase awareness of the risk factors identified here. This can be performed in a number of contexts other than driver training courses. For example, shift workers should be made aware of the increased risk of driving home after having worked a night shift. Indeed, a more holistic driving fatigue campaign is needed that addresses fatigue due to factors other than length of time spent driving. Many of these points may seem obvious, but they do not often form a major part of recommendations aimed at reducing road traffic incidents and injuries.^30^
